# Changes in the System of Nurse Training at the London Hospital

**Published:** 1918-05-25

**Authors:** 


*To the Editor of* THE HOSPITAL.

### Changes in the System of Nurse Training at the London Hospital.

*To the Editor of* THE HOSPITAL.

SIR,—In your article "The Making of a Trained Nurse" in THE HOSPITAL of May 4 you say that the London Hospital proposes to take a number of efficient V.A.D.s with one or two years' V.A.D. training, provided they undertake to go through a further two years' course as probationer nurses at the London Hospital; and further, that if such nurses pass successfully the usual test examinations held for ordinary probationers they shall be granted a three years' certificate at the end of a two years' course.

As two nurses from a London children's hospital we feel it our duty, in defence of those nurses who wish to take up further training at the end of their three years' course at a children's hospital, to point out that the V.A.D., excellent though she may be in many ways, never rises above the rank of probationer—that is, that though the sisters may have trusted her with some of the dressings and treatment if she has proved herself efficient, she has never been responsible for the management and routine of a ward. Then, again, much of the work done by ordinary probationers during their first year is done by hospital orderlies in the case of V.A.D. training. In our hospital, and so, presumably, in other children's hospitals, the probationers during their first year perform the same duties as we believe ordinary probationers in a general hospital do. Then, further, those nurses who have completed their three years' children's training and proved themselves efficient have been in charge of a ward as staff nurse both on night and day duty for the latter half of their training. In your article no mention is made of the London Hospital proposing to take nurses fully trained in a children's hospital for a two years' course with a three years' certificate, therefore we presume that the old rule is to hold good for them—*i.e.*, that if nurses with a three years' certificate from a recognised hospital for children wish to obtain a certificate at the London Hospital they must enter on a four years' course.

Surely it is absurd to imagine that anyone with a year's training as a V.A.D. can be as proficient as another who, in addition to a year or eighteen months' training as a probationer, has had a year and a half or two years as a staff nurse in charge of a ward of several children.

That being the case, why is it that the London Hospital, while proposing to give new facilities for further training to V.A.D.s, should not extend the same to nurses with a three years' certificate from a recognised hospital for children? We should very much like to know if the London Hospital contemplates extending the V.A.D. plan to admit children's nurses with a three years' certificate of training.—Yours obediently,

TWO NURSES TRAINING IN A HOSPITAL FOR CHILDREN.

